# Optimized path planning and scheduling strategies for connected and automated vehicles at single-lane roundabouts

**DOI:** 10.1371/journal.pone.0309732

**Published:** 2024-08-30

**Authors:** Lifeng Wang, Hu Liang, Yuxin Jian, Qiang Luo, Xiaoxiang Gong, Yiwei Zhang

**Affiliations:** 1 College of Mechanical Engineering, Chongqing Three Gorges University, Chongqing, China; 2 Key Laboratories of Sensing and Application of Intelligent Optoelectronic System, Sichuan University of Arts and Science, Sichuan, China; 3 Chongqing Engineering Research Center for Advanced Intelligent Manufacturing Technology, Chongqing Three Gorges University, Chongqing, China; 4 Chongqing Engineering Technology Research Center for Light Alloy and Processing, Chongqing Three Gorges University, Chongqing, China; University of Cincinnati, UNITED STATES OF AMERICA

## Abstract

This paper focuses on the cooperative driving challenges of connected and automated vehicles (CAVs) at single-lane roundabouts. First, a geometric path planning method is proposed for CAVs navigating a single-lane roundabout. Based on this method, a vehicle roundabout model is established. Four potential traffic scenarios for CAVs are established, and the optimal arrival times at conflict points are analyzed. By correlating the optimal arrival times at conflict points with the optimal entry times into the roundabout, the multi-vehicle coordination problem in complex intersections is simplified to a speed control issue during entry. Utilizing the principles of optimal control and Pontryagin minimization, two speed optimization strategies are proposed. Finally, MATLAB is employed for simulation analysis. The results indicate that the control strategy proposed in this paper enables the system to clearly identify potential conflicts between vehicles and implement an optimal control strategy, ensuring that vehicles can navigate the roundabout efficiently in terms of time and fuel without collisions. Additionally, the minimum time interval is established at 0.2 seconds to completely prevent vehicle collisions. In this study, the fusion problem involving two vehicles at a single conflict point is further expanded to encompass multiple vehicles at multiple conflict points. Thus, the efficient scheduling of multiple vehicles in single-lane roundabouts is realized.

## Introduction

**Fig 1:**
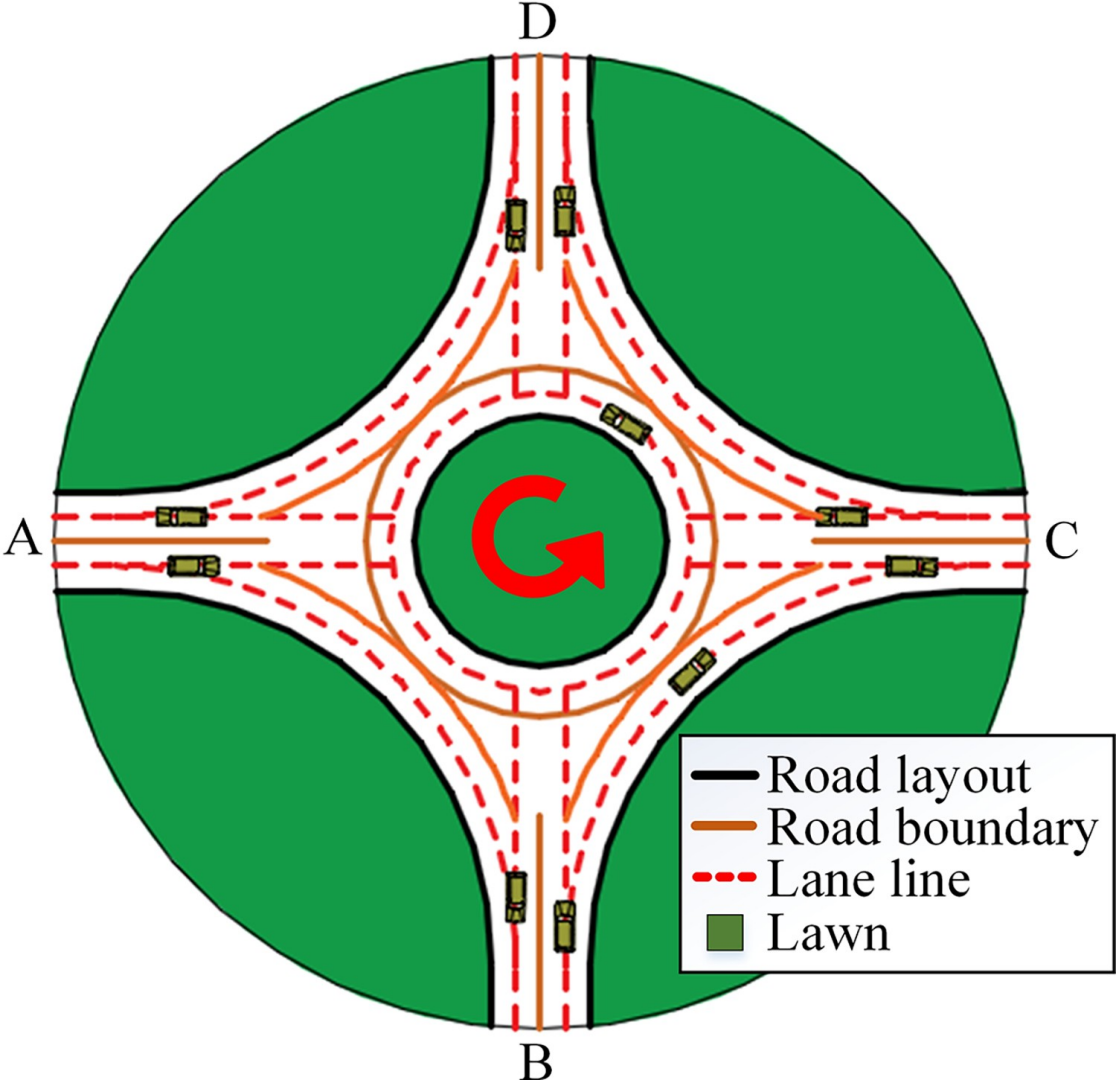
A typical single-lane roundabouts.

Roundabouts are utilized globally and recognized as a method to enhance traffic safety (Fig 1) [1]. Compared with signalized intersections, the number of conflict points is reduced, the conflict points are changed from intersection conflict to merging conflict, and the vehicle speed is reduced [2,3]. Therefore, roundabouts have lower accident rates and lower collision severity.

However, a significant drawback hampers the broader application of roundabouts: limited traffic capacity and unfamiliarity among drivers can result in increased delays and fuel consumption [4,5]. Typically, roundabout capacity can be enhanced through two approaches. The first method is a metering signal, which creates a gap for vehicles coming from the entrance lane by regulating the flow from the other entrance lane [6,7]. However, this method is effective primarily during peak periods when traffic flow diminishes significantly [8]. The second method is installing signals at all four entrances of a roundabout [9,10]. This approach transitions the roundabout’s operation from rule-based control to centralized management. However, it incurs substantial construction expenses and might compromise both safety and efficiency [9].

In recent years, the rapid development of connected and autonomous vehicles (CAVs), vehicle-to-vehicle (V2V) communications, advanced driver assistance systems (ADAS), and on-board sensing technologies has provided a possibility for improved data collection and vehicle control [11–15]. Among them, networked autonomous vehicles provide innovative solutions in path planning and scheduling control [16,17].

### Literature review

The application of CAVs to enhance roundabout capacity is a significant focus of current research [18–21]. P. Joshué et al. [22] proposed a control strategy for navigating autonomous vehicles in roundabouts. They defined the vehicle’s trajectory with a circular parametric equation; however, their path planning was confined to the roundabout. Other researchers have focused on the navigation control of individual autonomous vehicles at roundabouts [23,24]. Ali M et al. [25] proposed a real-time detection and navigation system for intelligent vehicles navigating urban roundabouts. This paper deals with single-vehicle scheduling at roundabouts but does not extend to multi-vehicle scenarios. Rastelli J P et al. [26] introduced a fuzzy logic-based steering control system for autonomous vehicles navigating roundabouts. Collectively, these studies focus on individual vehicles, neglecting the roundabout traffic as an integrated system. Several researchers have explored the scheduling of autonomous vehicles within roundabouts [27–29]. These researchers consider the roundabout as a comprehensive system, algorithmically managing the movement of each vehicle and utilizing a first-come, first-served method for conflict resolution [30]. However, this approach overlooks trajectory planning and conflict point analysis, leading to limitations in the comprehensive analysis of traffic systems. Fakirah M et al. [31] proposed a path planning for single vehicles in multi-lane roundabouts and developed an Intelligent Transportation System (ITS) [32]. However, the study focuses on a few typical steering issues without addressing the broader spectrum of vehicle dynamics or cooperative multi-vehicle scheduling. Ibanez G. et al. [33] identified collision points for vehicles in roundabouts and proposed strategies for collision avoidance. Hang P. et al. [34] utilized game theory to tackle the integration challenges faced by intelligent vehicles in roundabouts. Although the literature has theoretically explored numerous issues, the practical application of these complex algorithms continues to pose challenges. Bakibillah A S M et al. [35] introduced a bi-level control strategy for CAVs at roundabouts that enhances traffic flow and safety through vehicle coordination. However, this strategy assumes full CAV penetration and overlooks potential communication delays. The author also proposed a roundabout control system (RCS) to facilitate smooth traffic; however, the complexity of traffic scenarios and the necessity for real-time computing may limit the applicability of this strategy across different conditions [36]. Yao et al. [37] investigated the effects of CAV platoon sizes in mixed traffic scenarios using both analytical and simulation approaches. This paper presented a systematic method for assessing traffic flow efficiency; however, it may not adequately consider diverse driver reactions and unforeseen disruptions in traffic patterns. Rodrigues M et al. [38] proposed an adaptive tactical behavior planning method for autonomous vehicles navigating unsignaled intersections. It effectively addresses the safety and efficiency problems of merging but does not do research on multi-vehicle scheduling. In conclusion, roundabout traffic problems should be addressed as a comprehensive system, requiring integrated path planning and effective intersection conflict management.

### Contribution of the paper

In summary, previous studies have not succeeded in improving roundabout efficiency or reducing fuel consumption under different transportation demands. A centralized optimization method for CAVs applicable to single-lane roundabouts remains elusive. Thus, this paper aims to investigate the path planning and scheduling control for CAVs at single-lane roundabouts. The primary contributions of this paper are as follows:

1. A path planning method of CAVs for single lane roundabouts based on geometric solutions is proposed.
2. Four traffic scenarios that the autonomous vehicle may face at the roundabout are proposed, and the best time for the autonomous vehicle to reach the conflict point in different scenarios is analyzed.
3. The optimal time for vehicles to arrive at a series of conflict points is mapped to the optimal time for vehicles to enter the roundabout, and the complex multi-vehicle coordination problem of the roundabout is transformed into the problem of controlling vehicles to enter the roundabout at the optimal time.
4. Based on the optimal time and pontryagin minimum principle (PMP), two speed optimization modes are proposed. At the same time, the problem of rear-end collisions between vehicles on the same road is solved, which can-not be solved by the principle of minimum.
5. The fusion problem between two vehicles in the conflict point is extended to the fusion problem of multiple vehicles in the conflict point, so that the scheduling of multiple vehicles in the ring island is realized.

## Problem description and methodology

**Fig 2:**
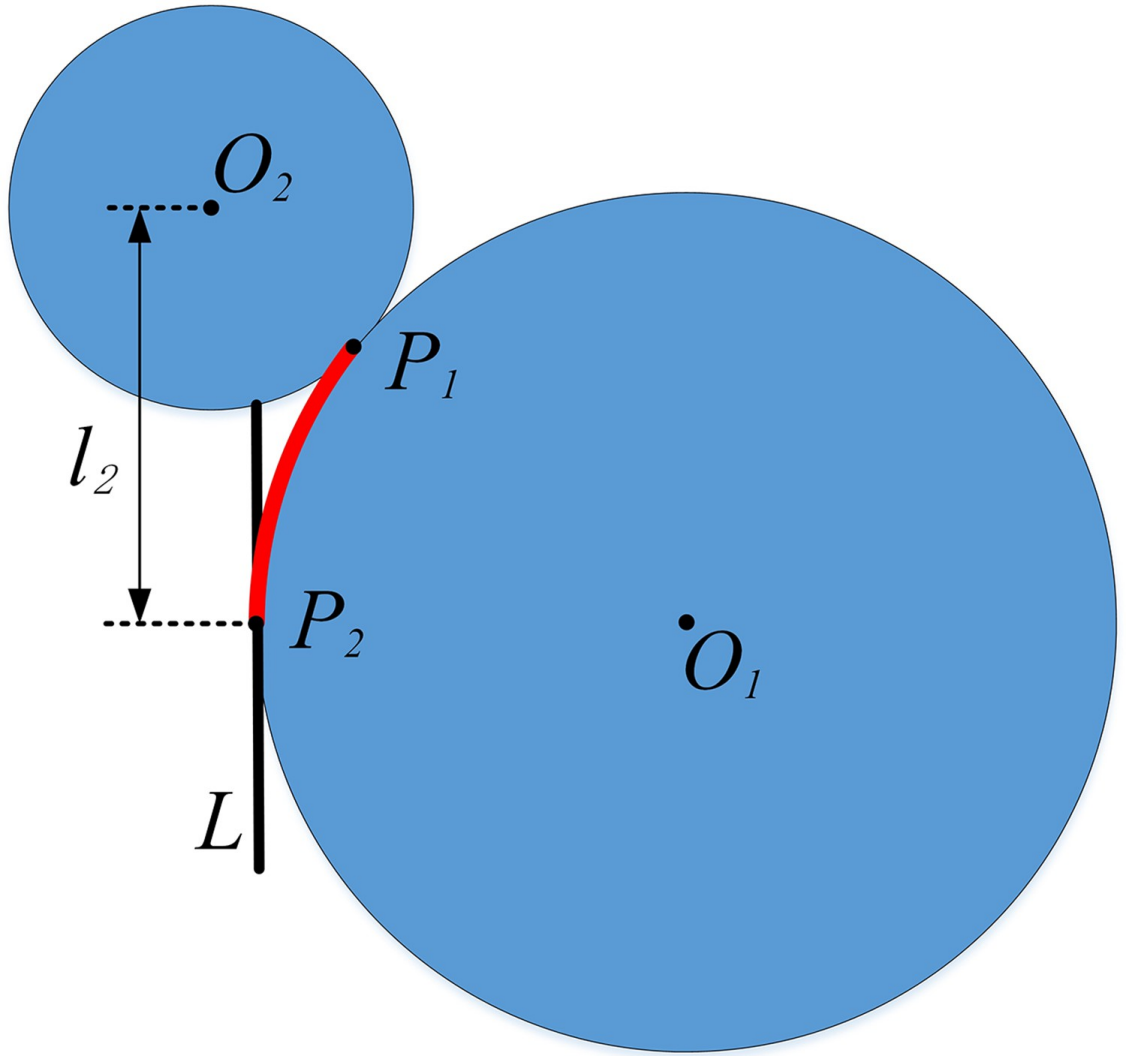
Geometric programming method.

Collaborative scheduling of CAVs within a roundabout necessitates path planning to identify the conflict point and the optimal entry route. This paper employs a geometric planning method to chart the path (Fig 2). Circles 1 and 2 are tangent to point *P*_*1*_, and circle 1 is tangent to line *L* at point *P*_*2*_. The arc *P*_*2*_*P*_*1*_ represents the desired path. Point *P*_*1*_ serves as both the confluence and conflict point.

**Fig 3:**
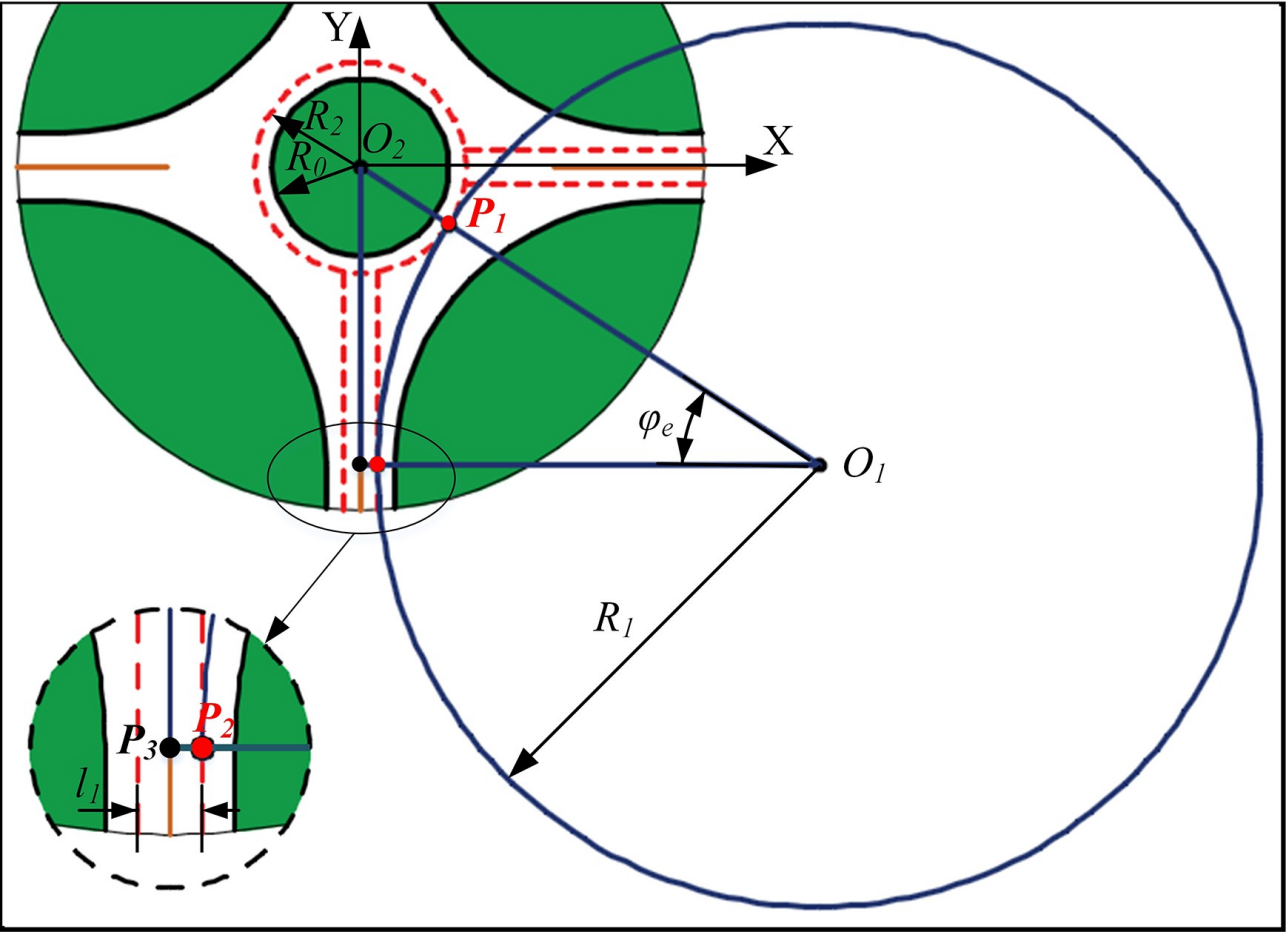
Geometric parameter diagram.

According to the geometric relationship (Fig 3), the following formula can be determined:

$$R_{1}=\frac{l_{1}^{2}+l_{2}^{2}-R_{2}^{2}}{2R_{2}-2l_{1}}$$ (1)

$$\varphi_{e}=\mathrm{atan}(\frac{l_{2}}{R_{1}+l_{1}})$$ (2)

$$ArcP_{2}P_{1}=\varphi_{e}R_{1}$$ (3)

Where *R*_*1*_ is the radius of the circumscribed circle of the required path, *l*_*1*_ is half the width of the road, *l*_*2*_ is the distance from the conflict point to the center of the roundabout, *φ*_*e*_ is the radian between the entry point and the conflict point, and *ArcP*_*2*_*P*_*1*_ is the arc length between the entry point and the conflict point.

### General system description

Under ideal conditions, in order to focus on the research of cooperative driving algorithms, some reasonable assumptions are made for all CAVs.

**Fig 4:**
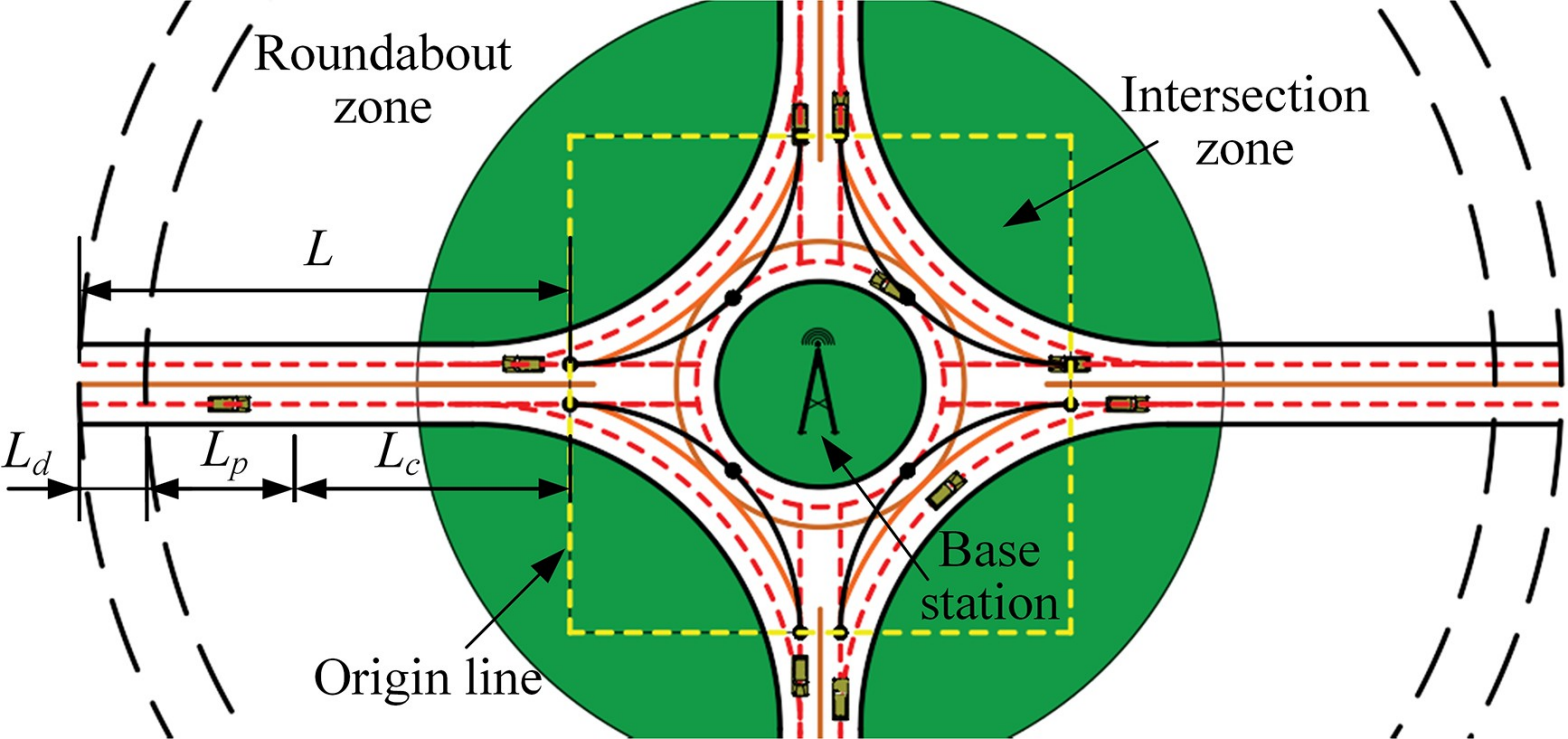
Roundabout layout with CAVs.

1. The roundabout and its surrounding areas are equipped with base stations that enable communication among all CAVs.

2. Good communication is between the base station and the vehicle, so this paper ignores the communication delay and packet loss of V2V and V2I.

3. CAVs can obtain their own information through sensors and obtain road information through base station sharing and other vehicle information. The details are as follows:

(1) The length of the takeover area near the roundabouts, including the length of the detection area, pre-control area, control area, and intersection area, as shown in Fig 4.

(2) The ID information of all vehicles is known, including the vehicle entry lane, exit lane, and arrival sequence.

(3) The real-time velocity and displacement of CAVs.

(4) The speed plan of CAVs to reach the collision point and the estimated time of reaching the collision point.

(5) Send an emergency signal when necessary.

4. All vehicles are fully driverless.

### Roundabout layout

Given the limited control range of vehicles within the roundabout, the paper proposes coordinating the scheduling of multiple vehicles to gain sufficient space. The proposed method extends the control range of vehicles traveling within the roundabout to areas outside the roundabout. The area adjacent to the roundabout is designated as the ’takeover area’ (Fig 4). Based on the varying functions needed by vehicles in different locations, the takeover area is further divided into three zones: a detection area, a pre-control area, and a control area, according to their proximity to the intersection. Specifically, *L*_*d*_ represents the detection area, *L*_*p*_ signifies the pre-control area, *L*_*c*_ stands for the control area, and *L* denotes the entire takeover area.

In the detection area, CAVs exchange information rapidly via base stations. Control strategies for CAVs are based on both their own information and the information from other CAVs. In the pre-control area, adjusting the speeds of following vehicles is essential to prevent rear-end collisions with preceding vehicles. Within the control area, each CAV must maintain a specified speed and reach the intersection zone within the allotted time. CAVs exit the roundabouts while maintaining a constant speed. For clarity, we designate the combined junction and intersection areas as the roundabout zone, with the CAVs’ entry point to the ring intersection referred to as the origin line.

### Vehicle constraints

To simplify the control process, we solely consider the longitudinal motion of vehicles within the takeover zone. For instance, overtaking and lane changes are prohibited. Consequently, all vehicles can be modeled as second-order linear systems, represented by the following expression:

$$\dot{x}=[\begin{array}{c} \dot{d}\\ \dot{v} \end{array}]=Ax+Bx=[\begin{array}{cc} 0 & 1\\ 0 & 0 \end{array}][\begin{array}{c} d\\ v \end{array}]+[\begin{array}{c} 0\\ 1 \end{array}]u$$ (4)

Where *d*, *v*, and *u* are the displacement, velocity, and acceleration of the CAVs, respectively.

1. Maximum acceleration and deceleration

Excessive longitudinal acceleration and deceleration adversely affect the comfort of passengers and drivers. Additionally, the extreme values of acceleration and deceleration must remain within the actuator capacity limits. Therefore, the constraints on vehicle input are defined as follows:

$$u_{\min}\leq u\leq u_{max}$$ (5)

Where *u* is the acceleration or deceleration of the CAVs, *u*_min_ and *u*_max_ are the maximum deceleration and acceleration of the CAVs.

2. Maximum speed of entering the roundabouts

Excessive longitudinal speed can lead to a loss of vehicle control, especially during tight turns at roundabouts. Although the minimum principle-based optimization algorithm cannot continuously account for state constraints, imposing speed limits at the end of the control zone effectively restricts vehicle speeds within that zone due to its spatial limitations. The speed constraints are defined as follows:

$$v_{f}=v_{f\max}$$ (6)

Where *v*_*f*_ is the speed of CAVs entering the roundabouts, *v*_*fmax*_ is the maximum speed. In this paper, it is set as 10m/s. When the CAVs enter the roundabouts, the speed should keep at 10m/s.

3. Minimum time interval at the conflict points

Muller E.R. et al. [39] proposed a criterion stating that the time difference between vehicles with potential conflicts traversing a conflict point on a given route must be greater than or equal to a minimum safety time interval. This approach minimizes the computational cost of coordinating operations among multiple vehicles. The proposed criteria are adopted in this study.

**Fig 5:**
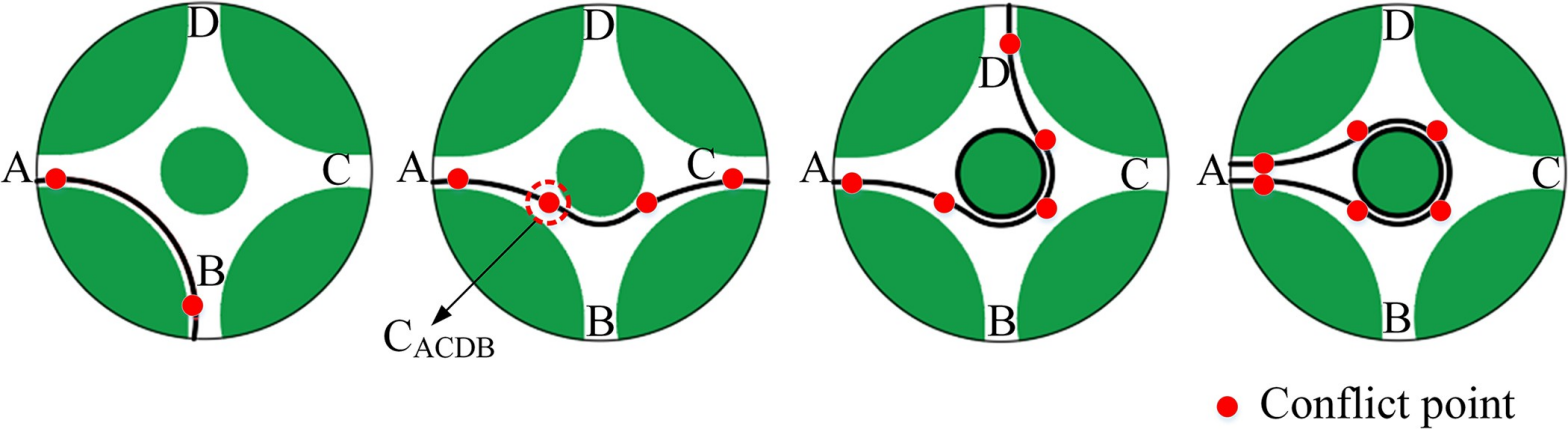
Conflict model.

To facilitate the analysis, we have marked the vehicle and roundabout models. Fig 5 illustrates that *P*_*AB*_ is the vehicle’s path in the roundabout zone, with A and B indicating the entrance and exit directions, respectively. The conflict point in the roundabout can be represented as *C*_*ACDB*_, specifically, the intersection formed by paths *P*_*AC*_ and *P*_*DB*_. A vehicle on path *P*_*AC*_ is denoted as *M* ^*i*^_*AC*_, where the superscript and subscript indicating the vehicle and the road section, respectively. The red dots indicate all potential conflict points along paths *P*_*AB*_, *P*_*AC*_, *P*_*AD*_, and *P*_*AA*_ (Fig 5).

In the roundabout area, the conflict behavior of multiple vehicles can be classified into two groups based on whether the potentially conflicting vehicles are in the same entrance lane. If two vehicles are driving in their respective control areas and occupying different lanes without interfering with each other, they are allowed to change positions before reaching the conflict point. If both the front and rear vehicles are traveling in the same entrance lane, the rear vehicle must follow the front vehicle to reach the common conflict point, thereby avoiding rear-end collisions. Consequently, two types of safety issues arise when the front and rear vehicles reach the conflict point:

$$\{\begin{array}{c} t_{ACDB}^{i}\geq t_{ACDB}^{i-1}+\Delta t\\ t_{ACDB}^{i}\leq t_{ACDB}^{i-1}-\Delta t \end{array}$$ (7)

$$t_{ACAB}^{i}\geq t_{ACAB}^{i-1}+\Delta t$$ (8)

Where *t*^*i*^_*ACDB*_ denotes the time when the CAV reached the point *C*^*i*^_*ACDB*_. *t*^*i-1*^_*ACDB*_ denotes the time of the previous CAV, and two CAVs don’t go in the same lane. *t* ^*i*^_*ACAB*_ denotes the time when the CAV reached the point *C*^*i*^_*ACAB*_. *t*^*i-1*^_*ACAB*_ denotes the time of the previous CAV, and two CAVs go in the same lane.

**Fig 6:**
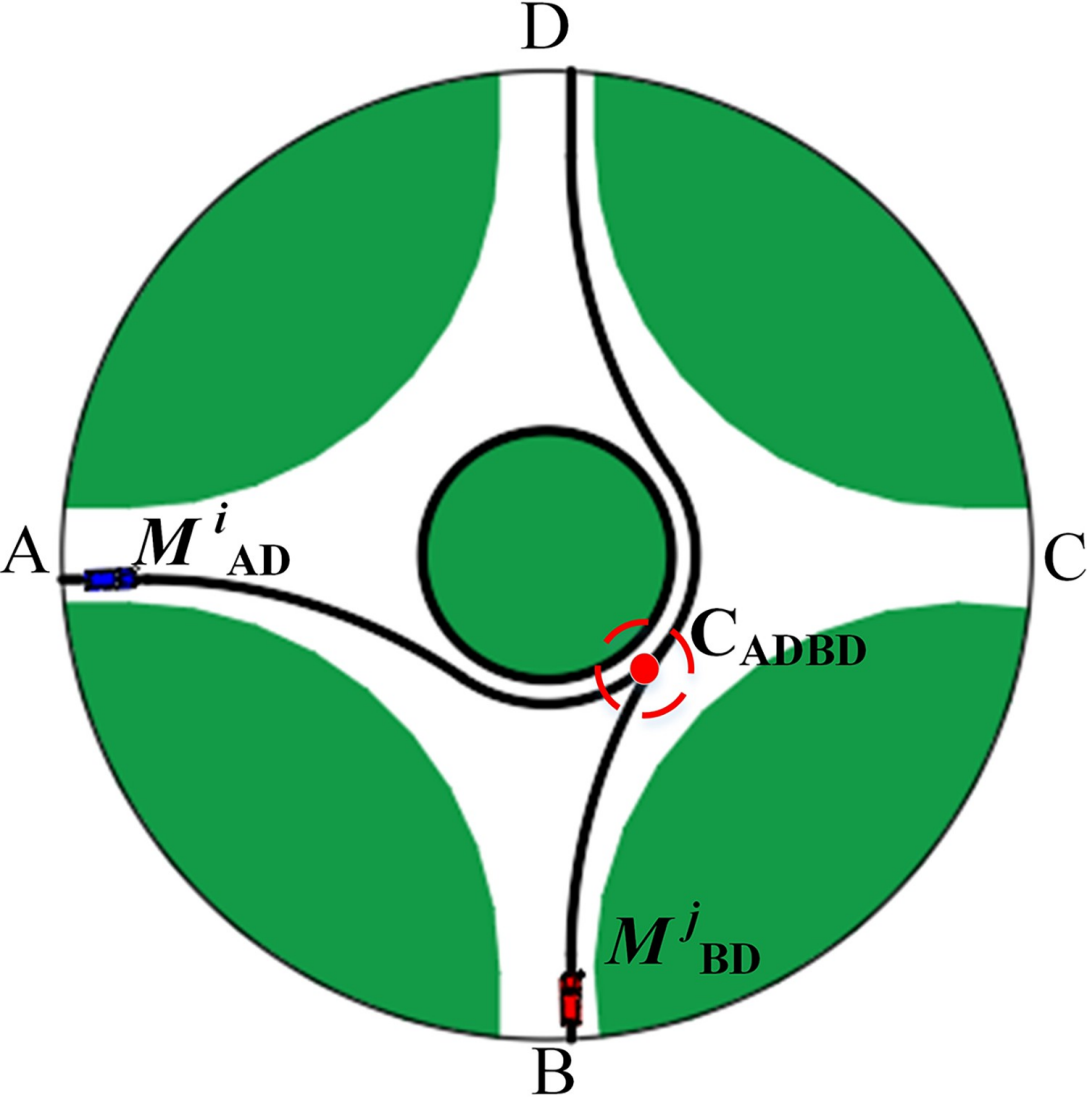
Conflict model II.

If the vehicle *M* ^*i*^_*AD*_ meets the vehicle *M* ^*i*^_*BD*_ at the conflict point *C*_*ADBD*_, a potential collision may occur (Fig 6). At this time, the vehicle *M* ^*i*^_*AD*_ has entered the roundabout at a speed of 10m/s. It is only necessary to ensure that the vehicle *M* ^*j*^_*BD*_ is ahead or behind *Δt* time interval. Refer to Eq 7.

## Cooperative control algorithm

### CAVs scheduling method

**Fig 7:**
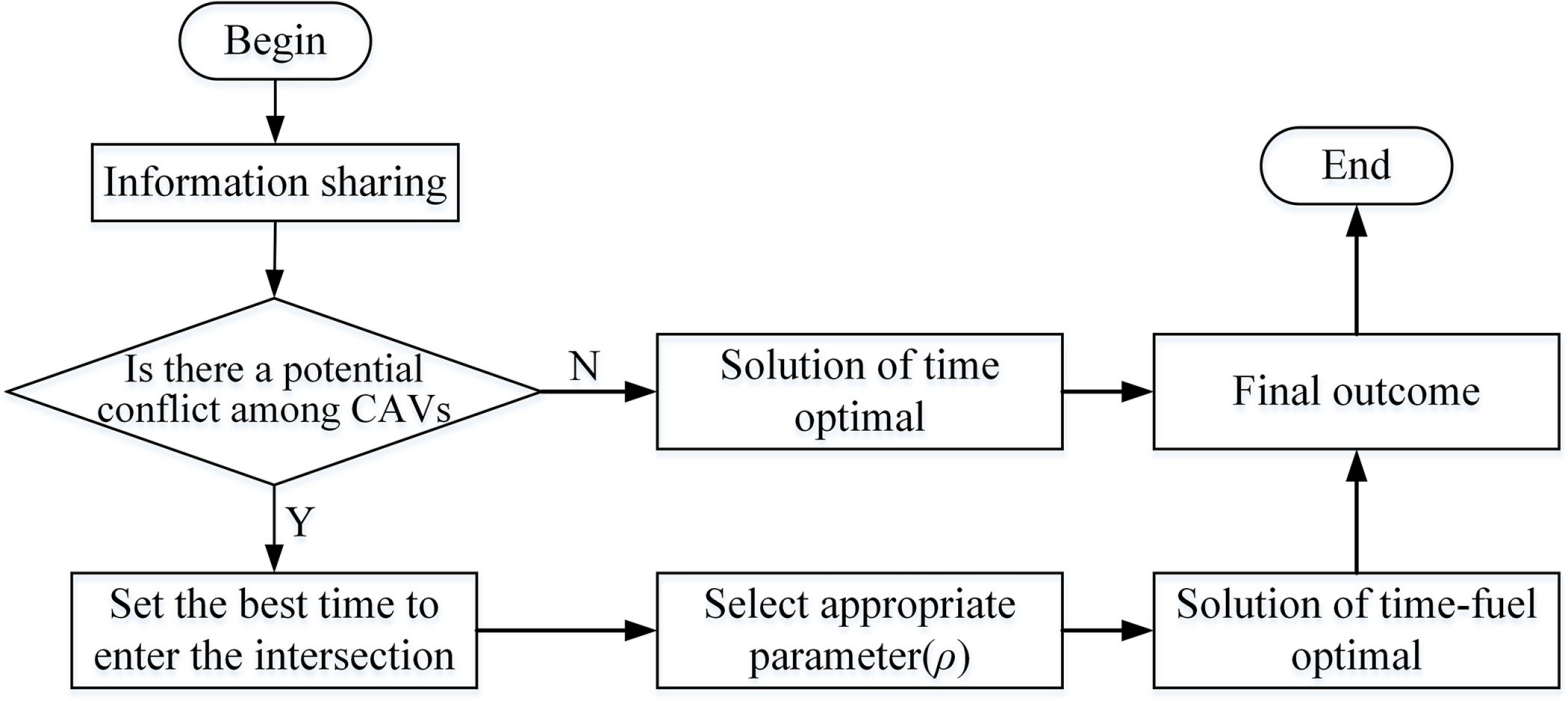
Procedure for generating a solution.

The concrete steps of vehicle cooperative scheduling at roundabouts based on the Pontryagin minimum principle adopted in this paper are shown in Fig 7. First, vehicles entering the pre-control area share information, and the system records their speed, displacement, serial numbers, and other relevant data. After entering the control area, vehicles proceed into the roundabout under optimal autonomous conditions. At this point, the system evaluates the time values of all vehicles entering monitored conflict points and then makes comparisons. If no two or more vehicles are present at the same conflict point simultaneously, the system directs the vehicle through the control area and into the roundabout using the optimal control method. If two or more vehicles arrive at the same conflict point simultaneously, the system identifies a conflict. The system then determines the serial numbers of the vehicles involved and identifies the rear vehicle. According to Formulas 7 and 8, it adjusts the rear vehicle’s running time to increase *Δt*. The system selects an appropriate *ρ* value and employs a time-fuel optimization scheme within the optimal control method to facilitate vehicle planning. Ultimately, vehicles navigate through the roundabout following the new plan. It is important to note that in a multi-vehicle collaborative environment, multiple conflicts may arise, necessitating the rapid and repeated application of the aforementioned methods for verification.

### Scheduling methods

**Fig 8:**
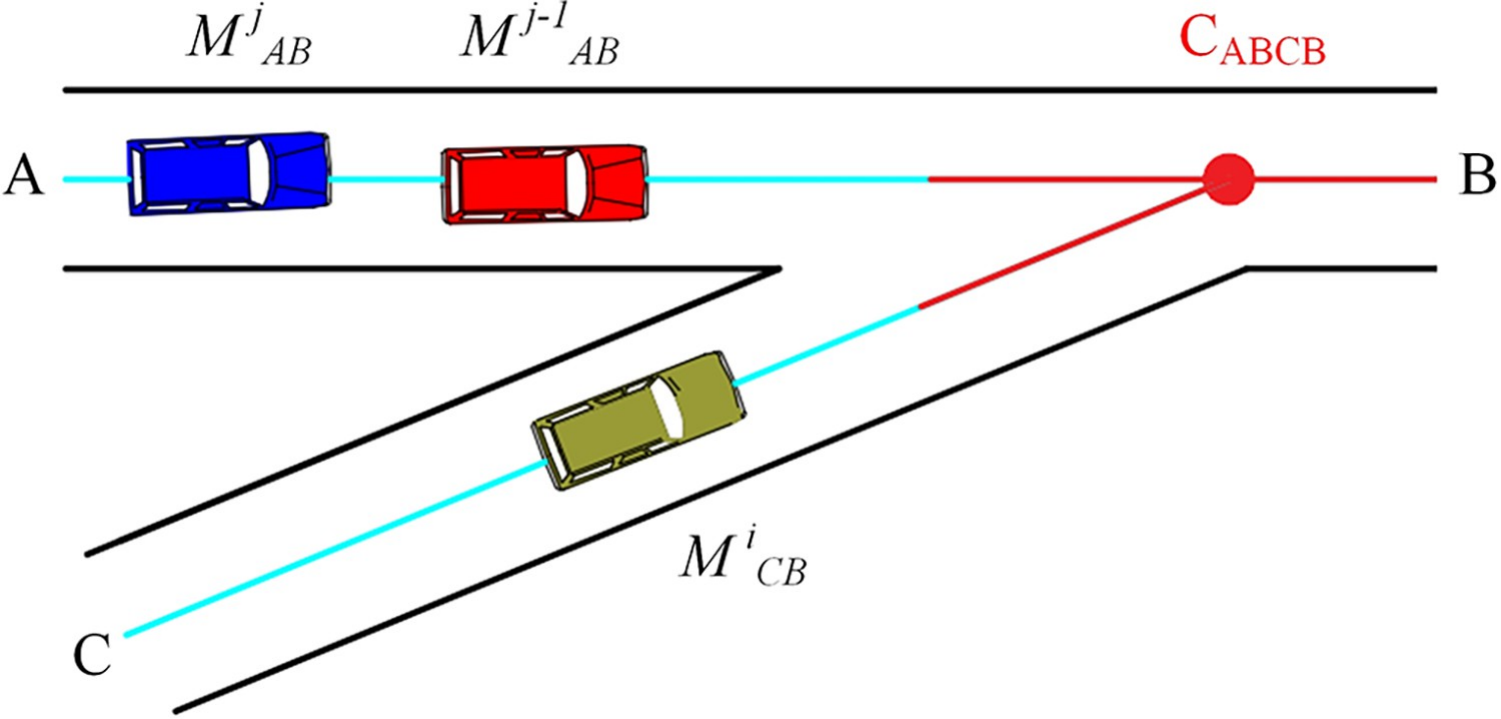
Model of single conflict point.

**Fig 9:**
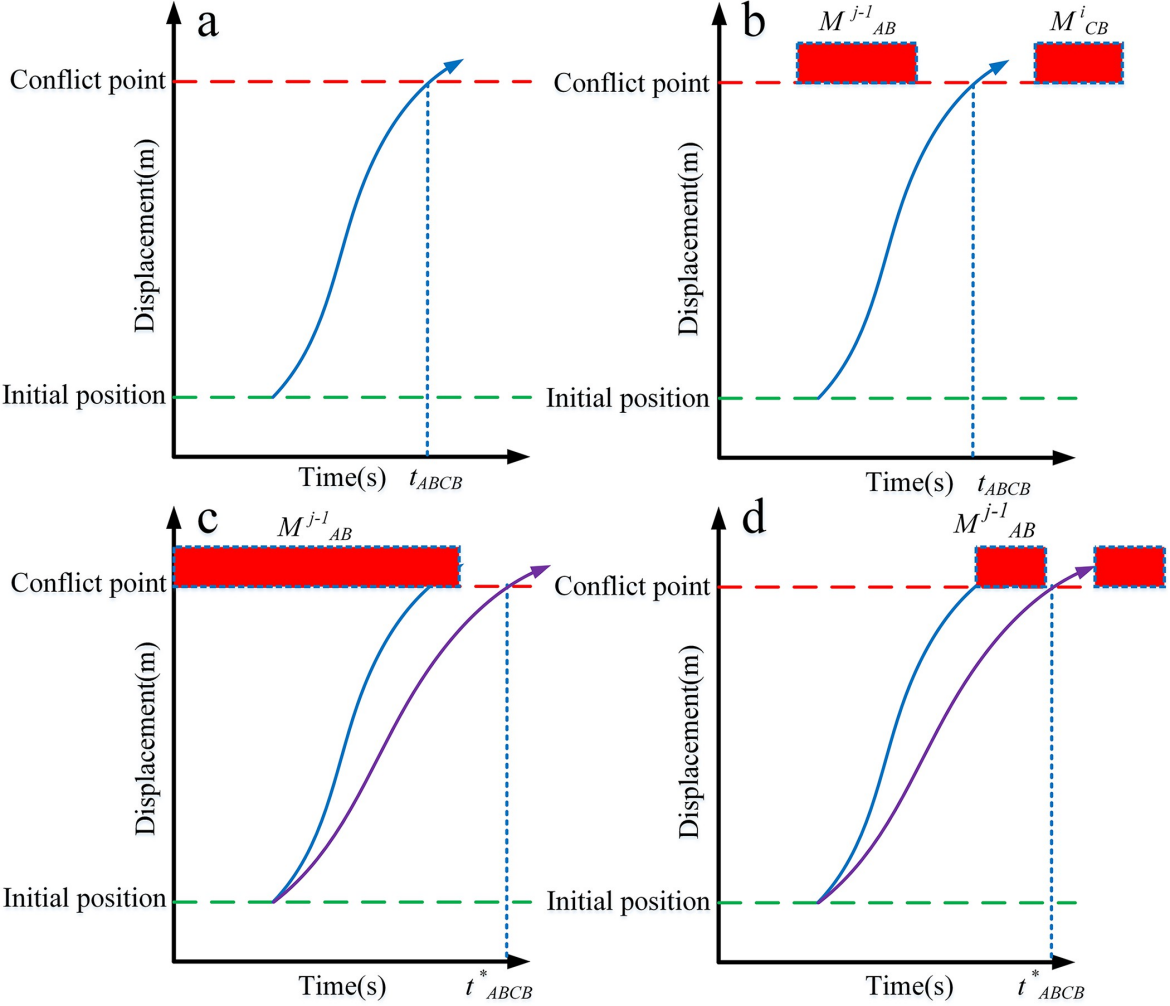
Search of reaching time for the four cases. (a) Case I. (b) Case II.(c) Case III. (d) Case IV.

The most critical and complex challenge in multi-vehicle cooperative driving around the island is to ensure safe passage for all vehicles. From a mathematical modeling perspective, this challenge involves determining the timing of vehicles as they reach a series of conflict points. The approach is outlined below:

This paper adopts the on-ramp confluence model to clearly describe the method of multi-vehicle cooperation (Fig 8). Fig 9 displays the relative positions of vehicle *M* ^*j*^_*AB*_ and other vehicles across four traffic scenarios and the time displacement diagram from the control area’s starting boundary to the conflict point. The blue and purple lines illustrate the time displacement curves of vehicle *M* ^*j*^_*AB*_ at the fastest and safest speeds, respectively, through the roundabout to the conflict point, while the red box indicates the time occupied by other vehicles (Fig 9).

Case I: In this scenario, the lack of interference from other vehicles prevents any collisions in the intersection area. The shortest time to the collision point *C*_*ABCB*_ corresponds to the optimal arrival time (Fig 9A).

$$t_{best}=t_{ABCD}$$ (9)

In this formula, *t*_*best*_ and *t*_*ABCD*_ express the optimal time and the fast time, respectively.

Case II: In this scenario, irrespective of whether vehicles are operating in the same or different lanes, the CAV can navigate the control zone at maximum speed without the risk of collision. This scenario corresponds to Formula (9) presented in Case I.

Case III: In this scenario, the leading vehicle *M* ^*j-1*^_*AB*_, traveling on the same road, restricts the trailing vehicle from reaching the conflict point at maximum speed. Since overtaking is not permitted, the trailing vehicle must remain behind the leading vehicle *M* ^*j-1*^_*AB*_ to reach the conflict point.

$$t_{best}=t_{ABCD}^{j-1}+△t$$ (10)

Where *t* ^*j-1*^_*ABCD*_ is the time for vehicle *M* ^*j-1*^_*AB*_ to reach the conflict point on the same road. The current vehicle arrives at the conflict point after the minimum safety time *Δt*, where t indicates that the current vehicle arrives at the conflict point.

Case IV: The previous three scenarios focus on roundabouts with sparse traffic or wide vehicle spacing. However, in reality, vehicles often congest and create busy conditions. Consequently, CAVs entering the roundabout encounter cross-lane interference, which makes it nearly impossible to navigate the control zone at optimal speed.

To simplify the computational complexity associated with multi-vehicle cooperative scheduling across multiple conflict points, we map the optimal arrival times for CAVs at various conflict points to the optimal entry times into the roundabout. The relationship between the CAVs’ arrival time at the conflict point and their entry time into the roundabout is delineated below:

$$t_{AB}^{E}=t_{ABCB}^{*}-\frac{D_{ABCB}}{v_{AB}^{E}}$$ (11)

Where *t*^*E*^_*AB*_ and t^*^_ABCB_ *are the* time to enter the roundabout zone and the time to reach the conflict point, respectively. *D*_*ABCB*_ is the displacement that the CAV runs along the path *P*_*AB*_ to *C*_*ABCB*_, *v*^*E*^_*AB*_ is the speed that the CAV leaves the control zone, and the value is 10m/s. The coordination problem of multiple vehicles at multiple conflict points in the roundabout zone is transformed into the problem of controlling vehicles to arrive at the roundabout zone at the right time.

### CAVs motion planning based on PMP

After assessing the detection zone, the autonomous vehicle begins to optimize its adjustments in the control area. In addition to ensuring absolute safety, the optimization primarily aims to enhance traffic flow and fuel efficiency. As a result, vehicles experience two speed optimization modes: time-fuel optimal and time-optimal.

Cases III and IV are time-fuel optimal models. Therefore, the cost function can be expressed as:

$$\min J=\int_{t_{e}}^{t_{f}}\left(\rho +|u|\right)dt$$

s.t.(5)and(6) (12)

Where *ρ* is the time coefficient and |*u*| is the absolute value of the control input. *t*_*f*_, *t*_*e*_ are the time instants for CAVs to enter and leave the control zone, respectively.

Considering Formulas (4) and (12), the Hamiltonian function can be generated as:

$$H(x,y,\lambda )=k+|u|+\lambda_{1}v+\lambda_{2}u$$ (13)

Where *λ*_*1*_, *λ*_*2*_ are the co-state components, respectively. The necessary condition for PMP is:

$$H[x^{*}(t),u^{*}(t),\lambda^{*}(t)]\leq \underset{u(t)\in \Omega }{H[x^{*}(t),u(t),\lambda (t)]}$$ (14)

Where x^*^(t) is the optimal state, u^*^(t) is the optimal input, and *Ω* is the allowable region of the control input.

According to Formulas (12) and (14), the optimal control input can be obtained as follows:

$$u^{*}(t)=\{\begin{array}{c} u_{\max},\lambda_{2}(t)<u_{\min}\\ 0,u_{\min}\leq \lambda_{2}(t)\leq u_{\max}\\ u_{\min},\lambda_{2}(t)>u_{\max} \end{array}$$ (15)

According to the analysis, the candidate control sequences are:{*u*_*max*_},{*u*_*min*_},{0,*u*_*max*_},{0,*u*_*min*_},{*u*_*max*_,0,*u*_*min*_},{*u*_*min*_,0,*u*_*max*_}.From the initial and final states of the vehicle in the control zone and the switching line rules of the minimum principle, it can be concluded that the optimal control sequence should be {*u*_*max*_,0,*u*_*min*_}.

Case I and Case II are time optimal mode. Therefore, the cost function is:

$$\min J=\int_{t_{e}}^{t_{f}}dt$$

s.t.(5)and(6) (16)

Analyzing the vehicle’s initial and final states within the control zone, along with the minimum principle’s switching line rules, indicates that the optimal control sequence is {*u*_*max*_, *u*_*min*_}.

### Rear-end collision avoidance

Although the proposed cooperative scheduling method successfully reduces the risk of multiple collisions at intersections, if the real-time speed of an autonomous vehicle exceeds that of the preceding vehicle before entering the control zone, a rear-end collision may occur in the entry lane, potentially leading to serious consequences. Therefore, it is crucial to implement preemptive control of the deceleration of autonomous vehicles in the pre-control zone. The equation for controlling the deceleration of autonomous vehicles in the pre-control zone is:

$$v_{AB}^{j}+(t_{AB}^{e}-t_{AB}^{s})u_{\min}=v_{AB}^{j-1}$$ (17)

Where *v* ^*j*^_*AB*_ is the speed at which vehicle *M* ^*j*^_*AB*_ enters the pre-control zone, *t* ^*e*^_*AB*_ is the time when vehicle *M* ^*j*^_*AB*_ leaves the pre-control zone. *t* ^*s*^_*AB*_ is the time when vehicle *M* ^*j*^_*AB*_ enters the pre-control zone, and *v* ^*j-1*^_*AB*_ is the speed of the vehicle *M* ^*j*^_*AB*_ in the same lane.

The vehicle *M* ^*j*^_*AB*_ should also meet the displacement requirements in the pre-control zone:

$$v_{AB}^{j}(t_{AB}^{e}-t_{AB}^{s})+\frac{1}{2}{\left(t_{AB}^{e}-t_{AB}^{s}\right)}^{2}u_{\min}=L_{p}$$ (18)

## Simulation

### Roundabout model

**Fig 10:**
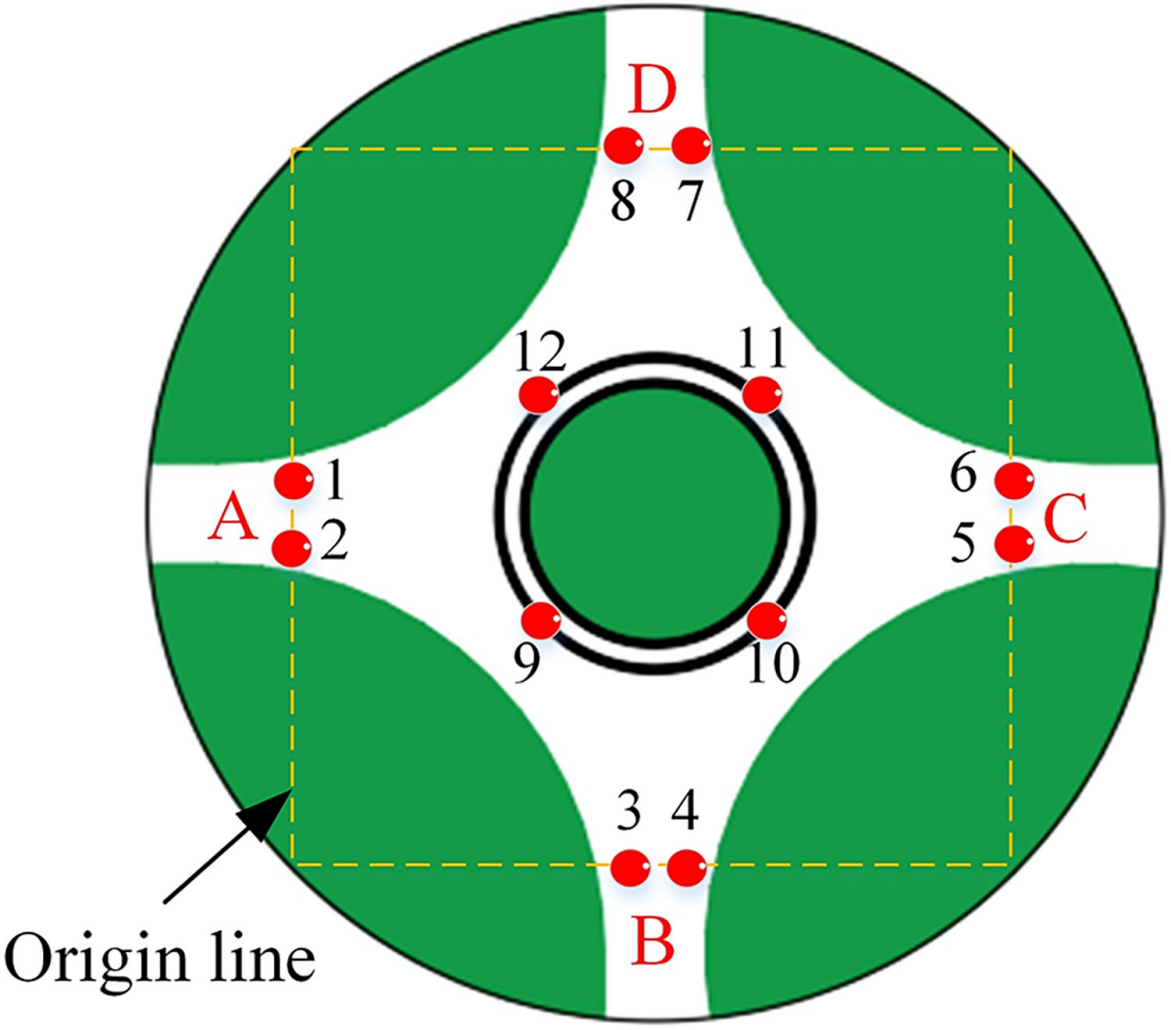
The layout of the roundabout on Kaizhou Road in Puyang City.

A MATLAB simulation (S1 File) is performed to validate the proposed algorithm’s effectiveness. The model simulates a real roundabout located on Kaizhou Road in Puyang, China. The model identifies 12 conflicting points, and the conflict points are numbered for clarity (Fig 10). The detecting, pre-control, and control zones measure 50 m (*L*_*d*_), 200 m (*L*_*p*_), and 500 m (*L*_*c*_) respectively.

Considering the roundabout’s symmetry, Table 1 shows the distance from the impact point to the origin line for departures in direction A.

**Table 1:** Distance from origin line to each conflict point from phase a.

No. of conflict point	1	2	3	4	5	6
**Distance(m)**	76.5	0	22.9	-	44.6	-
**No. of conflict point**	7	8	9	10	11	12
**Distance(m)**	61.5	-	13	30	47	63.5

### Traffic model

The constraint parameters *u*_*min*_ and *u*_*max*_ of CAV are set to -1m/s^2^ and 1m/s^2^ respectively. A roundabout simulation of three kinds of vehicle initial speed is designed.

Simulation I: All CAVs operate at high speeds with a minimum time interval of *Δt* = 0.2s. Table 2 displays the initial conditions for the high-speed CAV simulation.

Simulation II: All CAVs operate at variable speeds with a minimum time interval of *Δt* = 0.2s, as detailed in Table 3 for the mixed-speed simulation’s initial conditions.

Simulation III: All CAVs are operated at low speeds with a *Δt* = 0.2s minimum time interval. Table 4 presents the initial conditions for the low-speed CAV simulation.

**Table 2:** CAVs’ initial conditions for the high-speed simulation.

**Identifier**	***M***_***AB***_	***M***_***AC***_	***M***_***AD***_	***M***_***AA***_	***M***_***BA***_	***M***_***BC***_	***M***_***BD***_	***M***_***BB***_
***v***_***0***_ **(m/s)**	26	27	27.7	24	27.8	28	27	26.5
***t***_***0***_ **(s)**	2.3	5	1.5	5	0	2	8	2.5
**Identifier**	***M***_***CA***_	***M***_***CB***_	***M***_***CD***_	***M***_***CC***_	***M***_***DA***_	***M***_***DB***_	***M***_***DC***_	***M***_***DD***_
***v***_***0***_ **(m/s)**	26.5	27	27.8	25	27	27	28	28.5
***t***_***0***_ **(s)**	3.5	1	2.5	4	3	0	2.5	1

**Table 3:** CAVs’ initial conditions for the mixed-speed simulation.

**Identifier**	***M***_***AB***_	***M***_***AC***_	***M***_***AD***_	***M***_***AA***_	***M***_***BA***_	***M***_***BC***_	***M***_***BD***_	***M***_***BB***_
***v***_***0***_ **(m/s)**	27	15	25	15	13	8	27	15
***t***_***0***_ **(s)**	3	7	1.5	4.5	0	1	6	2.5
**Identifier**	***M***_***CA***_	***M***_***CB***_	***M***_***CD***_	***M***_***CC***_	***M***_***DA***_	***M***_***DB***_	***M***_***DC***_	***M***_***DD***_
***v***_***0***_ **(m/s)**	12	25	15	10	11	26	14	12.5
***t***_***0***_ **(s)**	3.5	2	3	4	1.5	0	2.5	1

**Table 4:** CAVs’ initial conditions for the low-speed simulation.

**Identifier**	***M***_***AB***_	***M***_***AC***_	***M***_***AD***_	***M***_***AA***_	***M***_***BA***_	***M***_***BC***_	***M***_***BD***_	***M***_***BB***_
***v***_***0***_ **(m/s)**	8	15	13	15	13	8	11	15
***t***_***0***_ **(s)**	3	7	1.5	4.5	0	1	6	2.5
**Identifier**	***M***_***CA***_	***M***_***CB***_	***M***_***CD***_	***M***_***CC***_	***M***_***DA***_	***M***_***DB***_	***M***_***DC***_	***M***_***DD***_
***v***_***0***_ **(m/s)**	12	13	15	10	11	15	14	12.5
***t***_***0***_ **(s)**	3.5	2	3	4	1.5	0	2.5	1

### Simulation result

Table 5 lists the arrival times of CAVs at various conflict points under the initial conditions of Situation I. Due to space constraints, schedules for other situations are not included.

**Table 5:** Time and minimum time interval when vehicles reach each conflict point for simulation I.

**Identifier**	***M***_***AB***_	***M***_***AC***_	***M***_***AD***_	***M***_***AA***_	***M***_***BA***_	***M***_***BC***_	***M***_***BD***_	***M***_***BB***_	***Δt***_***min***_ **(s)**
***t***_***1***_ **(s)**	-	-	-	47.1	38.7	-	-	-	0.7
***t***_***2***_ **(s)**	29.9	37.9	34.1	39.5	-	-	-	-	1.6
***t***_***3***_ **(s)**	32.1	-	-	-	-	-	-	43.5	3.1
***t***_***4***_ **(s)**	-	-	-	-	32.6	28.8	40.9	35.9	3.1
***t***_***5***_ **(s)**	-	42.4	-	-	-	31.1	-	-	1.2
***t***_***6***_ **(s)**	-	-	-	-	-	-	-	-	1.2
***t***_***7***_ **(s)**	-	-	40.3	-	-	-	45.4	-	0.7
***t***_***8***_ **(s)**	-	-	-	-	-	-	-	-	0.4
***t***_***9***_ **(s)**	-	39.2	35.4	40.8	-	-	-	42.2	0.6
***t***_***10***_ **(s)**	-	40.9	37.1	-	33.9	-	42.2	37.3	0.2
***t***_***11***_ **(s)**	-	-	38.6	44.2	35.6	-	43.9	38.9	0.3
***t***_***12***_ **(s)**	-	-	-	45.8	37.3	-	-	40.6	0.4
**Identifier**	***M***_***CA***_	***M***_***CB***_	***M***_***CD***_	***M***_***CC***_	***M***_***DA***_	***M***_***DB***_	***M***_***DC***_	***M***_***DD***_	***Δt***_***min***_ **(s)**
***t***_***1***_ **(s)**	41.1	-	-	-	32.5	-	-		0.7
***t***_***2***_ **(s)**	-	-	-	-	-	-	-		1.6
***t***_***3***_ **(s)**	-	40.1	-	-	-	37.4	-		3.1
***t***_***4***_ **(s)**	-	-	-	-	-	-	-		3.1
***t***_***5***_ **(s)**	-	-	-	45.6	-	-	41.2		1.2
***t***_***6***_ **(s)**	36.7	33.9	29.4	37.9	-	-	-		1.2
***t***_***7***_ **(s)**	-	-	31.7	-	-	-	-	41	0.7
***t***_***8***_ **(s)**	-	-	-	-	30.2	32.9	35	33.3	0.4
***t***_***9***_ **(s)**	-	38.6	-	42.6	-	35.9	38	36.3	0.6
***t***_***10***_ **(s)**	-	-	-	44.3	-	-	39.7	38	1.7
***t***_***11***_ **(s)**	38	35.2	-	39.2	-	-	-	40	0.3
***t***_***12***_ **(s)**	40	36.9	-	40.9	-	34.2	36.3	34.6	0.4

Note: *t*_*1*_ is the time when the CAV arrives at the conflict point c1, and *Δt*_*min*_ is the time interval.

**Fig 11:**
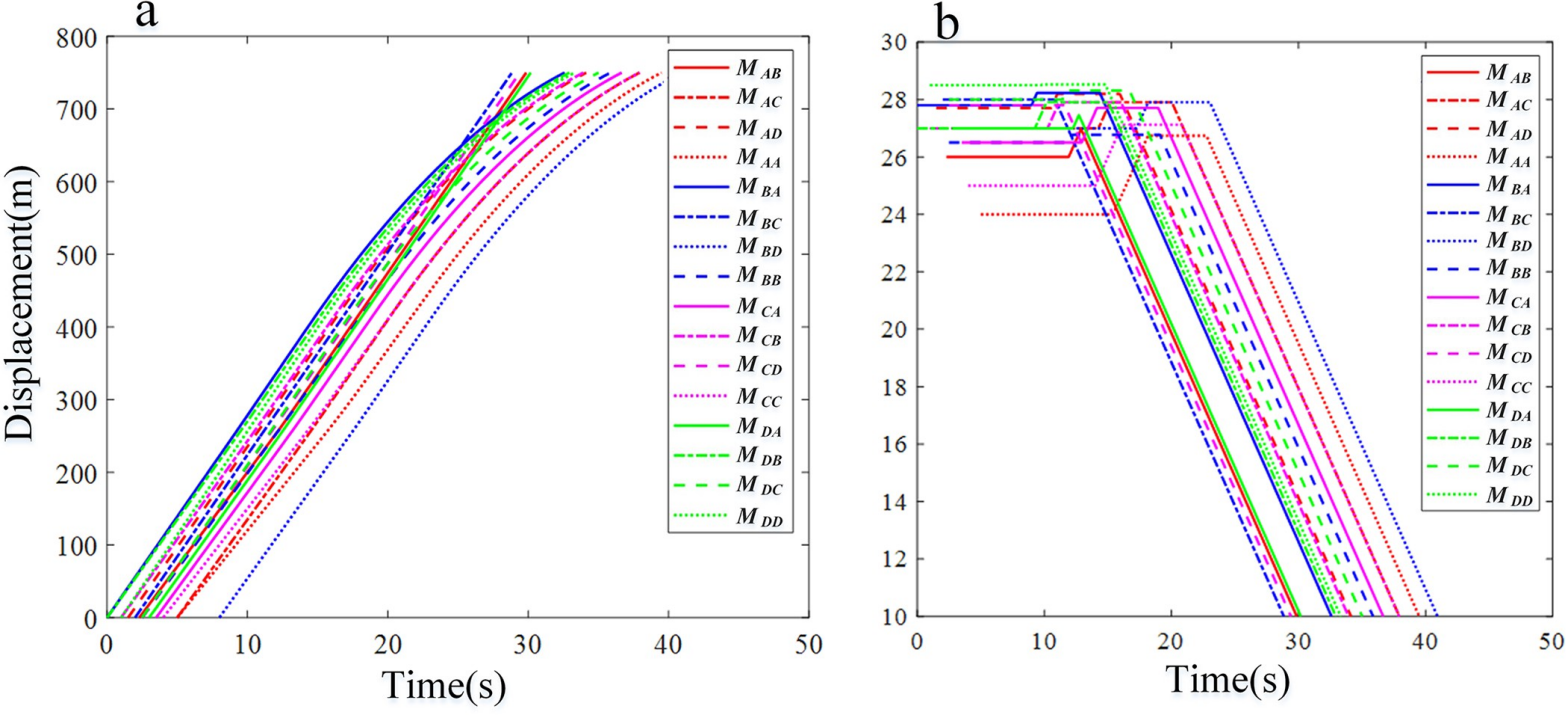
Analysis result curve of CAVs running in the takeover zone of Simulation I. (a) Displacement profiles. (b) Velocity profiles.

**Fig 12:**
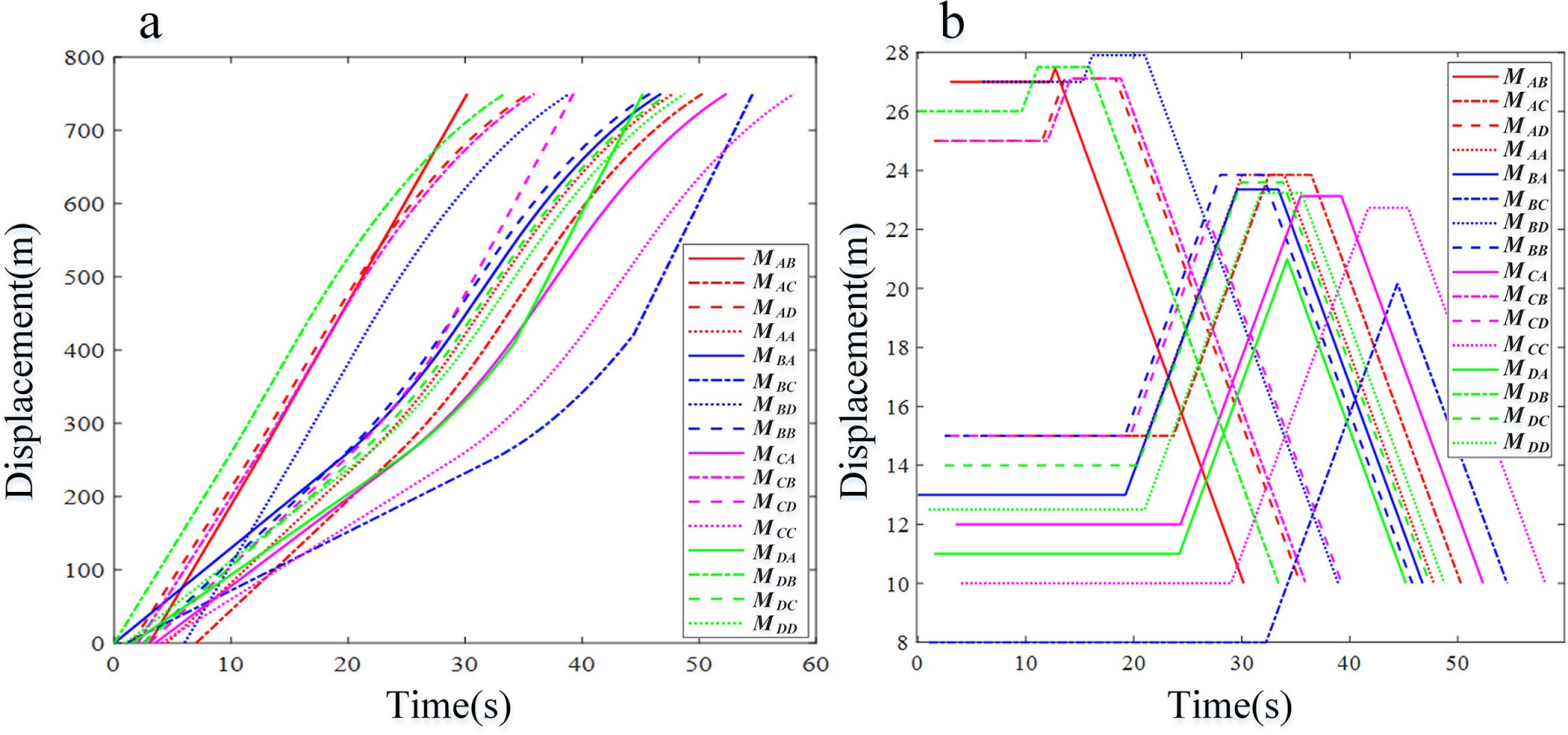
Analysis result curve of CAVs running in the takeover zone of Simulation II. (a) Displacement profiles. (b) Velocity profiles.

**Fig 13:**
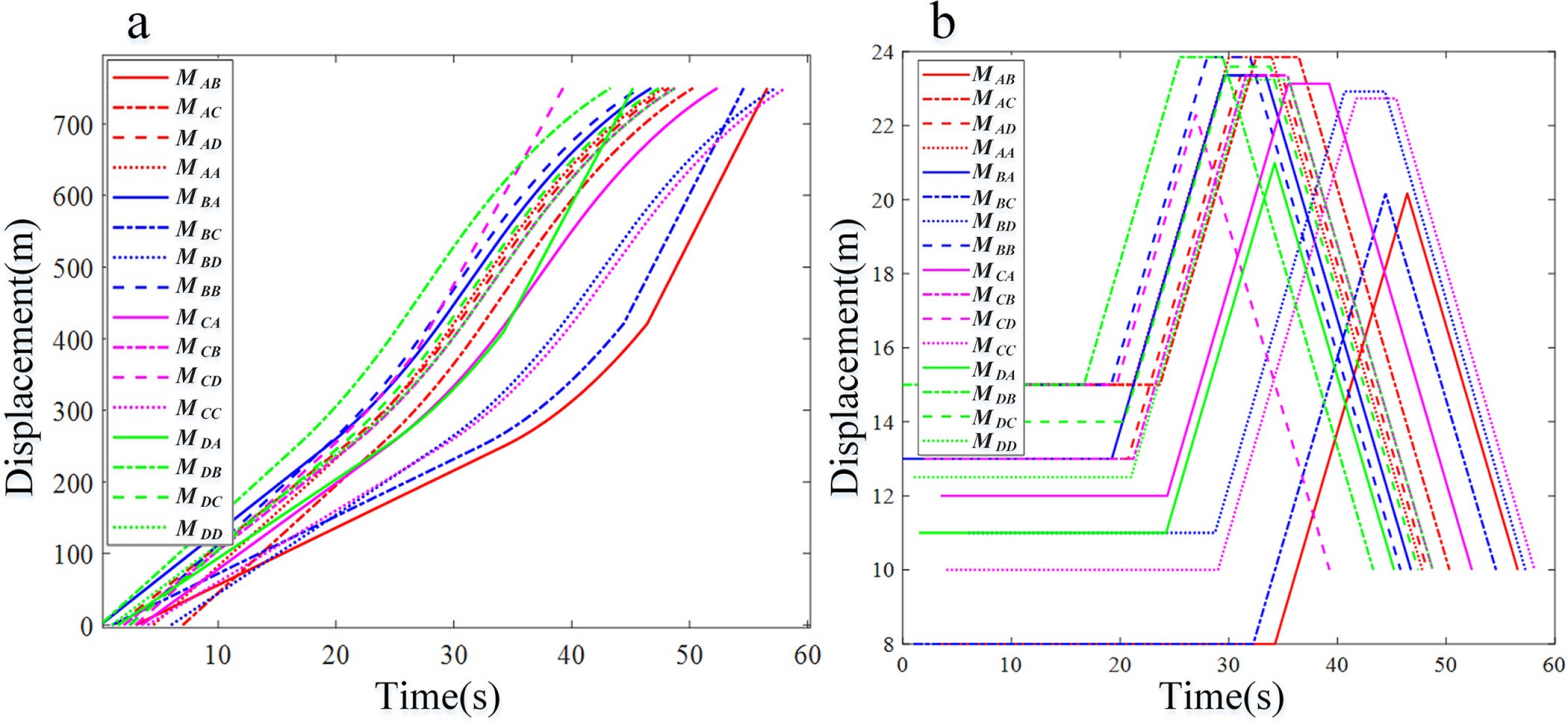
Analysis result curve of CAVs running in the takeover zone of Simulation III. (a) Displacement profiles. (b) Velocity profiles.

*M*_*AD*_ and *M*_*BB*_ are most likely to collide at conflict point 10 (Fig 11 and Table 5). The time interval of the vehicles is 0.2 seconds, which meets the requirements of the minimum time interval. The time interval for other vehicles is greater than the minimum safety standard, so all vehicles are able to pass without incident. All vehicles pass through the control area in a time-fuel optimal manner and traverse the roundabout at a constant speed (Fig 11B). Due to the mix of low- and high-speed vehicles, Fig 12A shows a more irregular distribution of vehicle displacement than Fig 11A. The vehicles *M*_*BC*_ and *M*_*AB*_ are in conflict, so the system re-plans and selects the time-optimal control strategy to pass through the control area and finally safely enter the roundabout. The vehicle displacement changes greatly at low speed, which means that the system needs more complex strategies when controlling low-speed vehicles and has a higher possibility of secondary planning (Figs 12 and 13).

Analysis of the three operating conditions indicates that without the control strategy proposed in this study, vehicle operation lacks clarity and may result in prolonged acceleration or deceleration. This not only wastes fuel and time but also compromises vehicle safety at conflict points. When utilizing the control strategy, the system can effectively identify potential conflicts and implement an optimal control approach, ensuring that vehicles navigate the roundabout efficiently in terms of time and fuel while avoiding collisions.

The analysis of fuel efficiency and compliance indicators reveals that CAVs *M*_*AC*_, *M*_*AD*_, *M*_*AA*_, *M*_*BA*_, *M*_*BD*_, *M*_*BB*_, *M*_*CA*_, *M*_*CB*_, *M*_*CC*_, *M*_*DB*_, *M*_*DC*_, and *M*_*DD*_ select the time-fuel optimal mode for navigating the roundabout area in complex scenarios (Fig 11). After optimizing the traffic efficiency index, priority should be given to maximizing fuel consumption efficiency. In addition, the roundabout multi-vehicle scheduling method based on the minimum principle proposed in this study is effective at different speeds, thus verifying its wide applicability (Figs 12 and 13). Given its low computational cost and comprehensive consideration of constraints, this method is proven to possess significant engineering application value.

**Fig 14:**
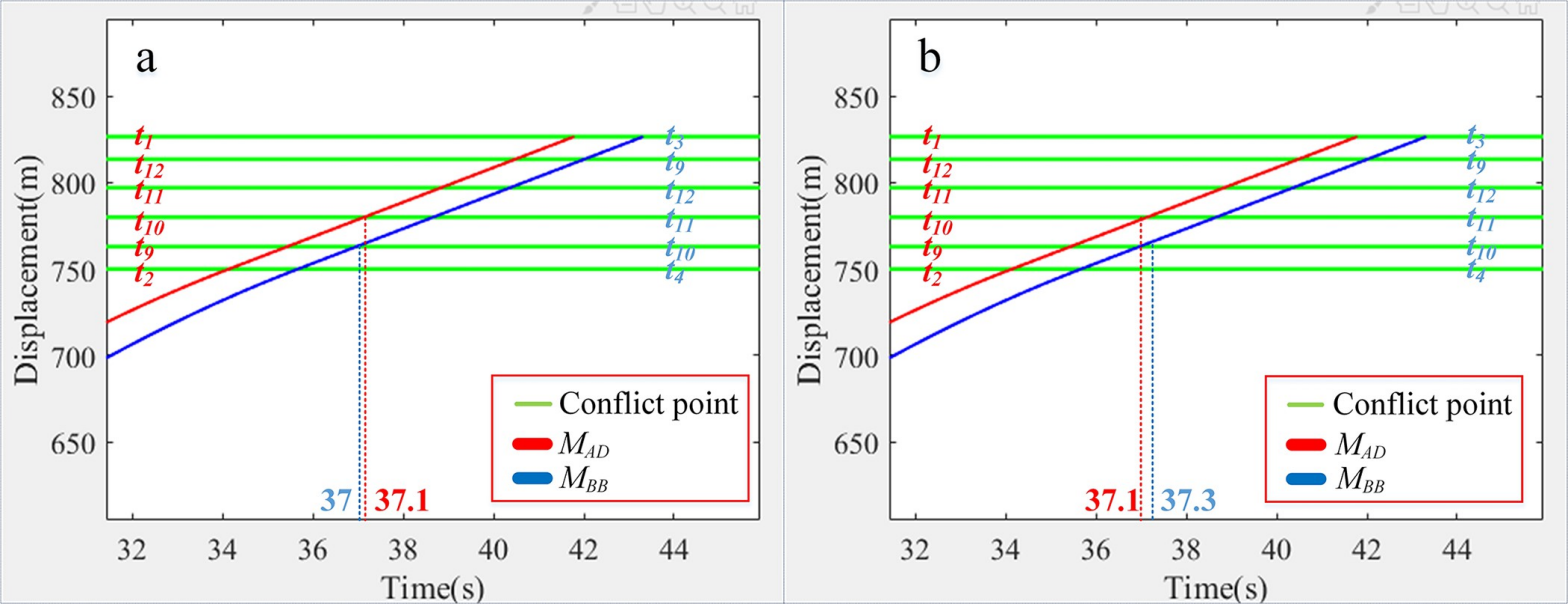
Comparison between *M*_*AD*_ and *M*_*BB*_ in Simulation I. (a) *Δt* = 0.1s. (b) *Δt* = 0.2s.

Table 5 presents the minimum intervals for each conflict point. Vehicles *M*_*AD*_ and *M*_*BB*_ are set to meet at t10 with a 0.1s interval, suggesting an imminent collision (Fig 14A). However, Eqs (7) and (11) establish a minimum interval of 0.2s, preventing any collision between the vehicles (Fig 14B). The last column of Table 5 confirms that all minimum intervals meet or exceed the safe interval of *Δt* = 0.2s, theoretically eliminating the risk of collisions using this algorithm.

## Conclusion and future work

A multi-autonomous vehicle cooperative driving method for single-lane roundabouts is proposed in this paper. First, due to the complexity of the path within the roundabout, the path planning process was simplified, and a geometric solution-based method for autonomous vehicle navigation in single-lane roundabouts was proposed, enhancing the conciseness and effectiveness of the analysis. Second, the vehicle roundabout model was constructed. Four typical traffic scenarios that an autonomous vehicle may encounter at a roundabout are then defined, along with an analysis of the optimal timing for reaching conflict points in these scenarios. Two speed optimization strategies are proposed by applying the principles of optimal control and Pontryagin minimization. These strategies optimize intersection timing, thereby enhancing the safety, speed, smoothness, and energy efficiency of the collaborative algorithm. Finally, MATLAB was employed for simulation analysis. The results demonstrate that the control strategy proposed in this study allows the system to clearly identify potential conflicts between vehicles and implement an optimal control strategy, ensuring that vehicles can navigate the roundabout in the most efficient manner regarding time and fuel without collisions. Additionally, when the interval between vehicles is 0.1 seconds, collisions may occur; therefore, the minimum interval is set to 0.2 seconds to completely prevent vehicle collisions.

In future research, we will focus on applying game-theory to control multi-vehicle intersections. We will study the macroscopic behavior of vehicles at roundabouts to enhance vehicle comfort and energy efficiency.

## Supporting information

S1 File (ZIP)
