# Supplementary material for: Optimized path planning and scheduling strategies for connected and automated vehicles at single-lane roundabouts
Source: PLoS One. 2024 Aug 30;19(8):e0309732. doi: 10.1371/journal.pone.0309732 (PMC11364289; doi:10.1371/journal.pone.0309732)
Supplement: S1 File — (ZIP) [file pone.0309732.s001.zip › S1 file/MATLAB program-Letgo.docx]

vad=27.7;

dtad=1.5;

%% Departing from direction A

d1=76.5;

d2=0;

d3=22.9;

d4=NaN;

d5=44.6;

d6=NaN;

d7=61.5;

d8=NaN;

d9=13;

d10=30;

d11=47;

d12=63.5;

%AA

[Dad Vad tad Tendad Dfad]=shuruTEnew(vad,dtad);

t1ad=Tendad+d1/10

t2ad=Tendad+d2/10

t9ad=Tendad+d9/10

t10ad=Tendad+d10/10

t11ad=Tendad+d11/10

t12ad=Tendad+d12/10

time=linspace(Tendad,t1ad,100);

weiyi=linspace(Dfad,d1+Dfad,100);

tad=[tad time];

Dad=[Dad weiyi];

vbb=26.5;

dtbb=2.5;

%% Departing from direction A

d1=61.5;

d2=NaN;

d3=76.5;

d4=0;

d5=22.9;

d6=NaN;

d7=44.6;

d8=NaN;

d9=63.5;

d10=13;

d11=30;

d12=47;

[Dbb Vbb tbb Tendbb Dfbb]=shuruTEnew(vbb,dtbb);

t3bb=Tendbb+d3/10

t4bb=Tendbb+d4/10

t10bb=Tendbb+d10/10

t11bb=Tendbb+d11/10

t12bb=Tendbb+d12/10

t9bb=Tendbb+d9/10

t3bb=Tendbb+d3/10

time=linspace(Tendbb,t3bb,100);

weiyi=linspace(Dfbb,d3+Dfbb,100);

tbb=[tbb time];

Dbb=[Dbb weiyi];

tf=t4bb+0.2-250/vbb-dtbb;

p=solveP(tf,vbb)

[Dbb Vbb tbb Tendbb Dfbb]=shuruTE1(vbb,dtbb);

t3bb=Tendbb+d3/10

t4bb=Tendbb+d4/10

t10bb=Tendbb+d10/10

t11bb=Tendbb+d11/10

t12bb=Tendbb+d12/10

t9bb=Tendbb+d9/10

t3bb=Tendbb+d3/10

time=linspace(Tendbb,t3bb,100);

weiyi=linspace(Dfbb,d3+Dfbb,100);

tbb=[tbb time];

Dbb=[Dbb weiyi];

% figure(1)

%

% plot(taa,Vaa,'r:','LineWidth',1.5);

% hold on

% xlabel('Time(s)','FontSize',25);

% ylabel('Longitudinal velocity(m/s)','FontSize',25);

% % axis equal;axis([-25,25,-25,25]);

% set(gca,'FontSize',15,'Fontname', 'Times New Roman');

figure(2)

plot([0,50],[750,750],'g','linewidth',2);

hold on

plot([0,50],[763,763],'g','linewidth',2);

hold on

plot([0,50],[780,780],'g','linewidth',2);

hold on

plot([0,50],[797,797],'g','linewidth',2);

hold on

plot([0,50],[813.5,813.5],'g','linewidth',2);

hold on

plot([0,50],[826.5,826.5],'g','linewidth',2);

hold on

plot(tad,Dad,'r','LineWidth',1.5);

hold on

plot(tbb,Dbb,'b','LineWidth',1.5);

hold on

xlabel('Time(s)','FontSize',25);

ylabel('Displacement(m)','FontSize',25);

% axis equal;axis([-25,25,-25,25]);

set(gca,'FontSize',15,'Fontname', 'Times New Roman');
