# Supplementary material for: Optimized path planning and scheduling strategies for connected and automated vehicles at single-lane roundabouts
Source: PLoS One. 2024 Aug 30;19(8):e0309732. doi: 10.1371/journal.pone.0309732 (PMC11364289; doi:10.1371/journal.pone.0309732)
Supplement: S1 File — (ZIP) [file pone.0309732.s001.zip › S1 file/MATLAB program-Run.docx]

%% Vehicle speed

v1=15;

v2=17;

v3=14;

v4=24;

v5=13;

v6=16;

v7=17;

v8=20;

v9=15;

v10=19;

%% Time of entering the detection area

dt1=0;

dt2=2;

dt3=0.8;

dt4=8;

dt5=1;

dt6=5.3;

dt7=4;

dt8=1.8;

dt9=3.9;

dt10=7;

%Time set for entering the detection area

B=[dt1,dt2,dt3,dt4,dt5,dt6,dt7,dt8,dt9,dt10];

%% Solving data

[D1 V1 t1 tf1]=shuruTE(v1,dt1);

[D2 V2 t2 tf2]=shuruT(v2,dt2);

[D3 V3 t3 tf3]=shuruTE(v3,dt3);

[D4 V4 t4 tf4]=shuruTE(v4,dt4);

[D5 V5 t5 tf5]=shuruTE(v5,dt5);

[D6 V6 t6 tf6]=shuruTE(v6,dt6);

[D7 V7 t7 tf7]=shuruT(v7,dt7);

[D8 V8 t8 tf8]=shuruTE(v8,dt8);

[D9 V9 t9 tf9]=shuruT(v9,dt9);

[D10 V10 t10 tf10]=shuruTE(v10,dt10);

figure(1)

plot(t1,V1);

hold on

plot(t2,V2);

hold on

plot(t3,V3);

hold on

plot(t4,V4);

hold on

plot(t5,V5);

hold on

plot(t6,V6);

hold on

plot(t7,V7);

hold on

plot(t8,V8);

hold on

plot(t9,V9);

hold on

plot(t10,V10);

hold on

figure(2)

plot(t1,D1);

hold on

plot(t2,D2);

hold on

plot(t3,D3);

hold on

plot(t4,D4);

hold on

plot(t5,D5);

hold on

plot(t6,D6);

hold on

plot(t7,D7);

hold on

plot(t8,D8);

hold on

plot(t9,D9);

hold on

plot(t10,D10);

%% Gather at the intersection time

A=[tf1,tf2,tf3,tf4,tf5,tf6,tf7,tf8,tf9,tf10];
