# Supplementary material for: Optimized path planning and scheduling strategies for connected and automated vehicles at single-lane roundabouts
Source: PLoS One. 2024 Aug 30;19(8):e0309732. doi: 10.1371/journal.pone.0309732 (PMC11364289; doi:10.1371/journal.pone.0309732)
Supplement: S1 File — (ZIP) [file pone.0309732.s001.zip › S1 file/MATLAB program-shuruT.docx]

function [D V t Tend]=shuruT(v,dt)

tt=250/v+dt;

a=5;

b=8*v-40;

c=2*v.^2-20*v-1000;

deta=b*b-4*a*c;

ta1=(-b+sqrt(deta))/2/a; %% The time at the turning point

ta2=(-b-sqrt(deta))/2/a;

T1=2*ta1+v-10; %%Total time spent

T2=2*ta2+v-10;

%% Velocity and displacement as functions of time

t3=linspace(dt,tt,50);

V3=v*ones(1,50);

D3=v*(t3-dt);

t1=linspace(0,ta1,100);

V1=t1+v;

D1=(1/2)*t1.^2+v*t1+v*(tt-dt);

t2=linspace(ta1,T1,100);

V2=-(t2-ta1)+v+ta1;

D2=-(1/2)*(t2-ta1).^2+(ta1+v)*(t2-ta1)+(1/2)*t2.^2+v*ta1+v*(tt-dt);

t=[t3 t1+tt t2+tt];

V=[V3 V1 V2];

D=[D3 D1 D2];

Tend=T1+tt;

end
