# Supplementary material for: Optimized path planning and scheduling strategies for connected and automated vehicles at single-lane roundabouts
Source: PLoS One. 2024 Aug 30;19(8):e0309732. doi: 10.1371/journal.pone.0309732 (PMC11364289; doi:10.1371/journal.pone.0309732)
Supplement: S1 File — (ZIP) [file pone.0309732.s001.zip › S1 file/MATLAB program-shuruTE.docx]

%%Vf, p unknown

function [D V t Tend]=shuruTE(v,dt)

tt=250/v+dt;

p=10;

vf=10;

a=2*p+4;

b=8*v+4*p*v;

c=p*v.^2+4*v^2-1000*p-(p+4)*vf.^2;

ta1=(-b+sqrt(b^2-4*a*c))/(2*a);

ta2=(-b-sqrt(b^2-4*a*c))/(2*a);

ta=ta1;

tb=(1000-2*ta*v-v.^2+vf.^2)/(2*v+2*ta);

tf=tb+ta+v-vf;

t0=linspace(dt,tt,100);

V0=v*ones(1,100);

D0=v*(t0-dt);

t1=linspace(0,ta,100);

V1=t1+v;

D1=(1/2)*t1.^2+v*t1+v*(tt-dt);

%

%

t2=linspace(ta,tb,100);

V2=(ta+v)*ones(1,100);

D2=(ta+v)*t2-(1/2)*ta.^2+v*(tt-dt);

%

%

t3=linspace(tb,tf,100);

V3=-t3+tb+ta+v;

D3=(-1/2)*(t3-tb).^2+(v+ta)*t3-(1/2)*ta.^2+v*(tt-dt);

%

t=[t0 t1+tt t2+tt t3+tt];

V=[V0 V1 V2 V3];

D=[D0 D1 D2 D3];

Tend=tf+tt;

end
