# Supplementary material for: Optimized path planning and scheduling strategies for connected and automated vehicles at single-lane roundabouts
Source: PLoS One. 2024 Aug 30;19(8):e0309732. doi: 10.1371/journal.pone.0309732 (PMC11364289; doi:10.1371/journal.pone.0309732)
Supplement: S1 File — (ZIP) [file pone.0309732.s001.zip › S1 file/MATLAB program-solveP.docx]

function p=solveP(tf,v)

%% tf is the optimal calculation time, and the time to remove the detection area p is the optimal time fuel ρ

vf=10;

a=2;

b=2.*(v-tf-vf);

c=1000-v.^2+vf.^2-2.*v.*tf+2*v.^2-2*v.*vf;

ta1=(-b+sqrt(b^2-4.*a.*c))./(2*a);

ta2=(-b-sqrt(b^2-4.*a.*c))./(2*a);

t=ta1;

p1=(4*vf.^2-4*v.^2-8*v*t-4*t.^2)./(2*t.^2+4*v*t+v.^2-1000-vf.^2);

t=ta2;

p2=(4*vf.^2-4*v.^2-8*v*t-4*t.^2)./(2*t.^2+4*v*t+v.^2-1000-vf.^2);

if p1>p2

p=p1;

else

p=p2;

end

end
