# Supplementary material for: Optimized path planning and scheduling strategies for connected and automated vehicles at single-lane roundabouts
Source: PLoS One. 2024 Aug 30;19(8):e0309732. doi: 10.1371/journal.pone.0309732 (PMC11364289; doi:10.1371/journal.pone.0309732)
Supplement: S1 File — (ZIP) [file pone.0309732.s001.zip › S1 file/MATLAB program-test.docx]

% v1=25;

% dt1=0;

%

% [D1 V1 t1 tf1]=shuruTE(v1,dt1);

% % [D2 V2 t2 tf2]=shuruT(v1,dt1);

%

% % tf1-tf2

% % tf=26.9757

%

% tf=tf1-100/v1;

% % tf=tf+2;

% p=solveP(tf)

% tf=26.8;

% tf1=28;

% tf2=29;

% tf3=30;

% tf4=31;

% tf5=32;

% tf6=33;

% A=[tf,tf1,tf2,tf3,tf4,tf5,tf6];

% p=solveP(tf);

% p1=solveP(tf1);

% p2=solveP(tf2);

% p3=solveP(tf3);

% p4=solveP(tf4);

% p5=solveP(tf5);

% p6=solveP(tf6);

% B=[p,p1,p2,p3,p4,p5,p6];

% plot(A,B)

% %% Different endpoint times correspond to different *ρ*

v1=26;

dt1=1;

[D1 V1 t1 tf1]=shuruTE(v1,dt1);

% [D2 V2 t2 tf2]=shuruT(v1,dt1);

% tf1-tf2

% tf=26.9757

tf=tf1-250/v1-dt1;

tf0=tf+0.2;

p=solveP(tf0,v1)

[D V t Tend]=shuruTE5(v1,dt1);

plot(t,V)

% [D V t Tend]=shuruTE5(v1,dt1);

% plot(t,V)
