# Supplementary material for: Optimized path planning and scheduling strategies for connected and automated vehicles at single-lane roundabouts
Source: PLoS One. 2024 Aug 30;19(8):e0309732. doi: 10.1371/journal.pone.0309732 (PMC11364289; doi:10.1371/journal.pone.0309732)
Supplement: S1 File — (ZIP) [file pone.0309732.s001.zip › S1 file/MATLAB program-workhighspeed.docx]

vab=26;

dtab=2.3;

vac=27;

dtac=5;

vad=27.7;

dtad=1.5;

vaa=24;

dtaa=5;

vba=27.8;

dtba=0;

vbc=28;

dtbc=2;

vbd=27;

dtbd=8;

vbb=26.5;

dtbb=2.5;

vca=26.5;

dtca=3.5;

vcb=27;

dtcb=1;

vcd=27.8;

dtcd=2.5;

vcc=25;

dtcc=4;

vda=27;

dtda=3;

vdb=27;

dtdb=0;

vdc=28;

dtdc=2.5;

vdd=28.5;

dtdd=1;

%% Departing from direction A

d1=76.5;

d2=0;

d3=22.9;

d4=NaN;

d5=44.6;

d6=NaN;

d7=61.5;

d8=NaN;

d9=13;

d10=30;

d11=47;

d12=63.5;

% AB

[Dab Vab tab Tendab]=shuruT(vab,dtab);

t2ab=Tendab

t3ab=t2ab+d3/10

% AC

[Dac Vac tac Tendac]=shuruTE(vac,dtac);

t2ac=Tendac

t5ac=Tendac+d5/10

t9ac=Tendac+d9/10

t10ac=Tendac+d10/10

%AD

[Dad Vad tad Tendad]=shuruTE(vad,dtad);

t2ad=Tendad+d2/10

t7ad=Tendad+d7/10

t9ad=Tendad+d9/10

t10ad=Tendad+d10/10

t11ad=Tendad+d11/10

%AA

[Daa Vaa taa Tendaa]=shuruTE(vaa,dtaa);

t1aa=Tendaa+d1/10

t2aa=Tendaa+d2/10

t9aa=Tendaa+d9/10

t11aa=Tendaa+d11/10

t12aa=Tendaa+d12/10

%% Departing from direction B

d1=61.5;

d2=NaN;

d3=76.5;

d4=0;

d5=22.9;

d6=NaN;

d7=44.6;

d8=NaN;

d9=63.5;

d10=13;

d11=30;

d12=47;

%BA

[Dba Vba tba Tendba]=shuruTE(vba,dtba);

t1ba=Tendba+d1/10

t4ba=Tendba+d4/10

t10ba=Tendba+d10/10

t11ba=Tendba+d11/10

t12ba=Tendba+d12/10

%BC

[Dbc Vbc tbc Tendbc]=shuruT(vbc,dtbc);

t4bc=Tendbc+d4/10

t5bc=Tendbc+d5/10

%BD

[Dbd Vbd tbd Tendbd]=shuruTE(vbd,dtbd);

t4bd=Tendbd+d4/10

t7bd=Tendbd+d7/10

t10bd=Tendbd+d10/10

t11bd=Tendbd+d11/10

%BB

[Dbb Vbb tbb Tendbb]=shuruTE(vbb,dtbb);

t3bb=Tendbb+d3/10

t4bb=Tendbb+d4/10

t9bb=Tendbb+d9/10

t10bb=Tendbb+d10/10

t11bb=Tendbb+d11/10

t12bb=Tendbb+d12/10

%111111111111111VBB needs to be+0.2 at t10

tf=t4bb+0.2-250/vbb-dtbb

p=solveP(tf,vbb)

%p=6.7

%% Starting from direction C

d1=44.6;

d2=NaN;

d3=61.5;

d4=NaN;

d5=76.5;

d6=0;

d7=22.9;

d8=NaN;

d9=47;

d10=63.5;

d11=13;

d12=30;

%CA

[Dca Vca tca Tendca]=shuruTE(vca,dtca);

t1ca=Tendca+d1/10

t6ca=Tendca+d6/10

t11ca=Tendca+d11/10

t12ca=Tendca+d12/10

%CB

[Dcb Vcb tcb Tendcb]=shuruTE(vcb,dtcb);

t3cb=Tendcb+d3/10

t6cb=Tendcb+d6/10

t9cb=Tendcb+d9/10

t11cb=Tendcb+d11/10

t12cb=Tendcb+d12/10

%CD

[Dcd Vcd tcd Tendcd]=shuruT(vcd,dtcd);

t6cd=Tendcd+d6/10

t7cd=Tendcd+d7/10

%CC

[Dcc Vcc tcc Tendcc]=shuruTE(vcc,dtcc);

t5cc=Tendcc+d5/10

t6cc=Tendcc+d6/10

t9cc=Tendcc+d9/10

t10cc=Tendcc+d10/10

t11cc=Tendcc+d11/10

t12cc=Tendcc+d12/10

%% D direction departure

d1=22.9;

d2=NaN;

d3=44.6;

d4=NaN;

d5=61.5;

d6=NaN;

d7=76.5;

d8=0;

d9=30;

d10=47;

d11=63.5;

d12=13;

%DA

[Dda Vda tda Tendda]=shuruT(vda,dtda);

t1da=Tendda+d1/10

t8da=Tendda+d8/10

%DB

[Ddb Vdb tdb Tenddb]=shuruTE(vdb,dtdb);

t3db=Tenddb+d3/10

t8db=Tenddb+d8/10

t9db=Tenddb+d9/10

t12db=Tenddb+d12/10

%DC

[Ddc Vdc tdc Tenddc]=shuruTE(vdc,dtdc);

t5dc=Tenddc+d5/10

t8dc=Tenddc+d8/10

t9dc=Tenddc+d9/10

t10dc=Tenddc+d10/10

t12dc=Tenddc+d12/10

%DD

[Ddd Vdd tdd Tenddd]=shuruTE(vdd,dtdd);

t7dd=Tenddd+d7/10

t8dd=Tenddd+d8/10

t9dd=Tenddd+d9/10

t10dd=Tenddd+d10/10

t11dd=Tenddd+d11/10

t12dd=Tenddd+d12/10

%BB

[Dbb Vbb tbb Tendbb]=shuruTE1(vbb,dtbb);

figure(1)

plot(tab,Vab,'r-','LineWidth',1.5)

hold on

plot(tac,Vac,'r-.','LineWidth',1.5);

hold on

plot(tad,Vad,'r--','LineWidth',1.5);

hold on

plot(taa,Vaa,'r:','LineWidth',1.5);

hold on

plot(tba,Vba,'b-','LineWidth',1.5);

hold on

plot(tbc,Vbc,'b-.','LineWidth',1.5);

hold on

plot(tbd,Vbd,'b:','LineWidth',1.5);

hold on

plot(tbb,Vbb,'b--','LineWidth',1.5);

hold on

plot(tca,Vca,'m-','LineWidth',1.5);

hold on

plot(tcb,Vcb,'m-.','LineWidth',1.5);

hold on

plot(tcd,Vcd,'m--','LineWidth',1.5);

hold on

plot(tcc,Vcc,'m:','LineWidth',1.5);

hold on

plot(tda,Vda,'g-','LineWidth',1.5);

hold on

plot(tdb,Vdb,'g-.','LineWidth',1.5);

hold on

plot(tdc,Vdc,'g--','LineWidth',1.5);

hold on

plot(tdd,Vdd,'g:','LineWidth',1.5);

hold on

xlabel('Time(s)','FontSize',25);

ylabel('Longitudinal velocity(m/s)','FontSize',25);

% axis equal;axis([-25,25,-25,25]);

set(gca,'FontSize',15,'Fontname', 'Times New Roman');

figure(2)

plot(tab,Dab,'r-','LineWidth',1.5)

hold on

plot(tac,Dac,'r-.','LineWidth',1.5);

hold on

plot(tad,Dad,'r--','LineWidth',1.5);

hold on

plot(taa,Daa,'r:','LineWidth',1.5);

hold on

plot(tba,Dba,'b-','LineWidth',1.5);

hold on

plot(tbc,Dbc,'b-.','LineWidth',1.5);

hold on

plot(tbd,Dbd,'b:','LineWidth',1.5);

hold on

plot(tbb,Dbb,'b--','LineWidth',1.5);

hold on

plot(tca,Dca,'m-','LineWidth',1.5);

hold on

plot(tcb,Dcb,'m-.','LineWidth',1.5);

hold on

plot(tcd,Dcd,'m--','LineWidth',1.5);

hold on

plot(tcc,Dcc,'m:','LineWidth',1.5);

hold on

plot(tda,Dda,'g-','LineWidth',1.5);

hold on

plot(tdb,Ddb,'g-.','LineWidth',1.5);

hold on

plot(tdc,Ddc,'g--','LineWidth',1.5);

hold on

plot(tdd,Ddd,'g:','LineWidth',1.5);

hold on

xlabel('Time(s)','FontSize',25);

ylabel('Displacement(m)','FontSize',25);

% axis equal;axis([-25,25,-25,25]);

set(gca,'FontSize',15,'Fontname', 'Times New Roman');
